# The association between maternal smoking and the risk of kidney diseases in offspring: A Mendelian randomization analysis based on large-scale GWAS

**DOI:** 10.1097/MD.0000000000045711

**Published:** 2025-11-07

**Authors:** Shumin Kang, Weidong Xu, Fengming Zhu, Zhenglong Zhang, Jian Zhang

**Affiliations:** a Pediatric Department, Zhangjiagang First People’s Hospital, Suzhou, Jiangsu, China.

**Keywords:** acute kidney injury, kidney diseases, maternal smoking, Mendelian randomization, offspring health, renal malignancies

## Abstract

Maternal smoking around birth (MSAB) has been associated with various adverse health outcomes in offspring, including an increased risk of kidney diseases. This study aims to investigate the potential causal relationship between MSAB and the risk of kidney diseases in descendants using Mendelian randomization (MR) methods, providing insights into the epidemiological background of kidney diseases and the application of MR in this context. We conducted a 2-sample MR analysis utilizing publicly available data from large-scale genome-wide association studies on MSAB (n = 397,732), breastfeeding (n = 352,094), and various kidney diseases, including acute kidney injury (AKI) (n = 482,266), chronic kidney injury (n = 482,858), glomerulonephritis (n = 500,348), renal malignancies (n = 463,010), and chronic kidney disease (n = 493,235). We employed multiple MR methods, including inverse variance weighted (IVW), weighted median, weighted mode, and MR Egger regression, to assess the causal effects. We used the odds ratio (OR) as our measure and conducted multivariable MR analysis to account for the confounding effect of breastfeeding. To ensure the reliability and stability of our results, we conducted sensitivity analyses, which included Cochran *Q* test, MR Egger, and leave-one-out analysis. The MR-IVW results indicated a strong positive causal association between MSAB and the risk of AKI (OR = 11.47, 95% confidence interval [CI]: 2.58–51.02, *P*-fdr = .008) and renal malignancies (OR = 1.02, 95% CI: 1.01–1.04, *P*-fdr = .01) in offspring. A potential causal relationship with glomerulonephritis was also observed (OR = 8.63, 95% CI: 1.13–65.88, *P*-fdr = .08). After adjusting for breastfeeding using multivariable MR, the associations between MSAB and AKI (OR: 13.43, 95% CI: 2.72–66.25, *P*_IVW_ = .001) as well as renal malignancies (OR: 1.02, 95% CI: 1.01–1.04, *P*_IVW_ = .005) remained statistically significant. This suggests that maternal smoking during pregnancy significantly increases the risk of AKI and renal malignancies in their children. Our study provides compelling evidence for a causal link between maternal smoking and an increased risk of kidney diseases in offspring, emphasizing the importance of addressing maternal smoking as a modifiable risk factor. Future research should focus on elucidating the underlying biological mechanisms and exploring interventions to mitigate the impact of maternal smoking on offspring health.

## 1. Introduction

Kidney disease is a growing global issue and is the 3rd leading cause of death worldwide.^[1]^ The burden of kidney disease is rising worldwide. According to the Global Burden of Disease study, global incident cases of chronic kidney disease (CKD) have more than doubled between 1990 and 2019.^[2]^ Research has shown that kidney disease is associated with adverse consequences in organs such as the heart, lungs, and brain.^[3]^ The dangers posed by kidney disease are extensive, impacting not only the kidneys but also multiple organ systems and the overall well-being of individuals. Therefore, it is crucial to identify the early risk factors associated with kidney diseases.

Maternal smoking around birth (MSAB) is a significant public health concern. It has substantial implications for the health of offspring. Epidemiological studies suggest that approximately 10% to 12% of pregnant women smoke around the world,^[4]^ and maternal smoking is associated with various adverse outcomes. The developmental origins of health and disease hypothesis posits that adverse environmental conditions during critical developmental periods can predispose individuals to various health issues.^[5]^ Addressing this issue is crucial because maternal smoking affects both immediate neonatal health and contributes to long-term health complications in offspring.^[6,7]^ Despite considerable attention, the specific impact of MSAB on kidney diseases in offspring remains unclear. De Smidt et al collected data at 5 years of age and demonstrated a reduction in kidney length in the smoking group during pregnancy compared to the control group.^[8]^ However, existing research has primarily focused on short-term effects, and evaluating long-term impacts could provide further insights into disease susceptibility in adult life.^[9]^

Research suggests that breastfeeding is associated with subclinical changes in childhood kidney outcomes. A prospective study measured the renal volumes in 5043 children in the Netherlands, among children with a median age of 6 years, compared with ever-breastfed children, never-breastfed children had smaller combined kidney volumes (−2.69 [95% confidence interval [CI], −4.83 to −0.56] cm^3^) and lower estimated glomerular filtration rates (−2.42 [95% CI, −4.56 to −0.28] mL/min/1.73 m^2^) at school age. Although the observed differences are not clinically significant, minor variations in kidney function at a young age may be linked to clinically relevant kidney outcomes in later life.^[10]^ While Escribano et al found that 6-month-old infants who were given high-protein infant formula had noticeably larger kidney volumes than those who were breastfed.^[11]^ Since these were conducted using observational studies, they are vulnerable to confounding biases. Thus definitive conclusions on whether breastfeeding can reduce the risk of kidney disease in offspring induced by MSAB remain unclear.

MR is a powerful approach that helps overcome some of the challenges posed by traditional observational studies by using genetic variants as instrumental variables to infer causality. This method leverages the random assortment of alleles during meiosis, which can reduce confounding and eliminate reverse causation.^[12]^ By utilizing genetic data, MR studies can provide more reliable estimates of the causal effects of maternal smoking on offspring health outcomes, offering insights that are less susceptible to biases commonly seen in observational studies. Given the significant public health implications of maternal smoking, employing MR is essential to better understand its impact on the health of future generations.

## 2. Materials and methods

### 2.1. Research design and data sources

**Figure 1.**
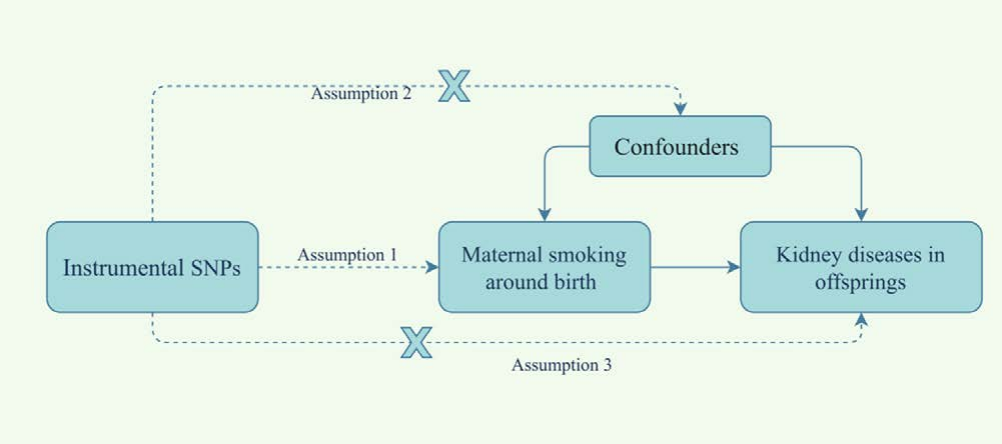
Model of the 2-sample Mendelian randomization analysis.

This study employs publicly available genome-wide association studies (GWAS) summary statistics for a MR analysis. The research design follows the STROBE-MR guidelines for reporting observational studies in epidemiology using Mendelian randomization (MR) (see STROBE-MR-checklist).^[13]^ The MR approach relies on 3 key assumptions (Fig. 1): first, the single nucleotide polymorphism (SNPs) acting as instrumental variables must be strongly linked to MSAB and meet genome-wide significance (Assumption 1). Second, these instrumental variables should be independent of confounding factors (Assumption 2). Third, they should influence the outcome only through the exposure, not through other pathways (Assumption 3).^[14]^

Genetic data were sourced from large-scale GWAS involving individuals of European ancestry. MSAB was used as the exposure variable in this study, which was identified by participants answering the question, “Did your mother smoke regularly around your birth?” The outcome variable was kidney disease, encompassing several conditions such as acute and chronic renal failure, glomerulonephritis, renal malignancies, CKD, membranous nephropathy, and tubulointerstitial disease. Breastfeeding was used as a covariate in the multivariate MR, which was confirmed by answering “Were you breastfed when you were a baby?” Relevant data for this study, including MSAB, breastfeeding status, acute and chronic renal failure, and renal malignancies, are available at https://gwas.mrcieu.ac.uk/. Additional data, encompassing glomerulonephritis and CKD, can be accessed at https://www.finngen.fi/fi. The GWAS data sources are described in detail in Table 1. Finnish Biobank and UK Biobank are large-scale biomedical databases and research resources. They contain genetic information and health data for approximately 500,000 Finnish and 500,000 UK participants.^[15,16]^ Disease endpoints (including the kidney diseases mentioned in the article) are mapped into phecode, a hierarchical grouping system of Electronic Health Record-based disease codes used for conducting phenome-wide association study, and International Classification of Diseases, 10th Revision, a medical classification list by the World Health Organization that is widely used for billing purposes. This mapping enables harmonized GWASs in UK Biobank and FinnGen.^[17]^

**Table 1:** Summary of GWAS data in this study.

Trait	GWAS ID	Year	Sample size	Number of SNPs	Population
Maternal smoking around birth	ukb-b-17685	2018	397,732	9851,867	European
Breastfed as a baby	ukb-b-13423	2018	352,094	9851,867	European
Acute renal failure	ebi-a-GCST90018790	2021	482,266	24,187,658	European
Chronic kidney disease	–	2024	493,235	20112651	European
Chronic renal failure	ebi-a-GCST90018822	2021	482,858	24,185,976	European
Glomerulonephritis	–	2024	500,348	20,112,714	European
Malignant neoplasm of kidney	ukb-b-1316	2018	463,010	9851,867	European

GWAS = genome-wide association study, SNP = single nucleotide polymorphism.

### 2.2. Selection of genetic instruments

Single nucleotide polymorphisms (SNPs) significantly associated with MSAB (*P* < 5 × 10⁻⁸) were selected as instrumental variables (IVs). To ensure the robustness of the instruments, SNPs were clumped based on linkage disequilibrium (*r*² < 0.001 within a 10,000 kb window). Only SNPs with a minor allele frequency > 0.01 were included to avoid bias from rare variants. The strength of the genetic instruments was assessed using the *F*-statistic calculated as (β^2^/SE^2^),^[18]^ with an *F*-statistic > 10 indicating strong instruments.^[19]^ Characteristics of the genetic variants associated with MSAB and breastfeeding can be found as Table S1, Supplemental Digital Content, https://links.lww.com/MD/Q561.

This study also employed LDTrait (a parallel alternative to Phenoscanner) available at [https://ldlink.nih.gov/?tab=home] to characterize all SNPs identified within the significant causal evidence, aiming to discern associations with MSAB. Since the smoking status of offspring may influence their risk of developing kidney disease, and the genetic variants rs10226228, rs36072649, rs6011779, and rs75596189 are all associated with current smoking behavior, these SNPs are considered potential confounding factors. Therefore, excluding these SNPs reduces the direct association between instrumental variables and confounding factors, thereby minimizing confounding bias.^[20]^

### 2.3. MR analysis

The primary analysis was conducted using the inverse variance weighted (IVW) method, which estimates the causal effect of an exposure on an outcome by aggregating individual SNP-specific associations through an IVW meta-analysis. This approach prioritizes SNPs with higher precision by using a regression model that forces the intercept to zero, which implicitly assumes the absence of horizontal pleiotropy.^[21,22]^ It relies on the validity of instrumental variable assumptions which means that the SNPs must be strongly associated with the exposure, independent of confounders, and exhibit no direct effects on the outcome. Therefore, Cochran *Q* test was used to assess heterogeneity among the genetic variants.^[23]^ Moreover, we assessed all identified heterogeneous SNPs responsible for possible unbalanced horizontal pleiotropy using LDTrait. MR Egger detects and adjusts for directional pleiotropy by incorporating an intercept term in the regression. A non-zero intercept indicates unbalanced pleiotropy, while the slope provides a pleiotropy-corrected causal estimate.^[24]^ Weighted median (WM) robustly estimates causality by taking the median of SNP-specific effects weighted by their precision. Valid even if up to 50% of SNPs violate instrumental variable assumptions.^[25]^ The WM method categorizes SNPs by their similarity using estimates of individual proportions and calculates the variance weighted count for each group. Ultimately, it derives a causal estimate according to the group of SNPs by the largest weighted number.^[26]^ Leave-one-out analysis was performed to evaluate whether the results were driven by any single SNP.

### 2.4. Multivariable MR (MVMR)

A MVMR analysis is particularly useful in cases where a standard MR analysis would fail due to violation of assumptions IV2 and IV3. It is also useful in cases where 2 or more correlated exposures are of interest and may help to understand whether both exposures exert a causal effect on the outcome, or whether one in fact mediates the effect of the other on the outcome.^[27]^

To address potential confounding by breastfeeding, a MVMR analysis was performed. This approach adjusted for both MSAB and breastfeeding at the same time, enabling the estimation of the independent causal effect of maternal smoking on kidney disease outcomes. The MVMR analysis was conducted using summary statistics from GWAS datasets that included information on both MSAB and breastfeeding.^[28]^

SNPs used as instrumental variables in multivariable MR should meet the following conditions: SNPs are associated with all exposure factors in the model; they do not affect the outcome variables in other ways; and the number of SNPs is greater than the number of exposure factors.^[29]^

All analyses were performed using R software (version 4.3.3; The R Foundation for Statistical Computing, Vienna, Austria) with the TwoSampleMR. The results are expressed as odds ratios (OR) and 95% CI.^[30,31]^ All IVW results were adjusted for multiple testing using the false discovery rate (FDR) method. A *P*-value < .05 was considered statistically significant for the primary analysis.

## 3. Results

### 3.1. Association between MSAB and offspring kidney diseases (prior to smoking-related SNPs removal)

The results of MR analysis(Table 2) showed that before removing offspring-related smoking SNPs, MSAB increased the appearance of acute renal failure in the offspring by 12.18-fold (OR = 13.18, 95% CI: 3.86–45.04, *P*-fdr < .001), increased the risk of CKD by 1.82-fold (OR = 2.82, 95% CI: 1.06–7.51, *P*-fdr = .05), increased the risk of chronic renal failure by 4.21-fold (OR = 5.21, 95% CI: 1.76–15.44, *P*-fdr = .009), and increased the risk of malignant neoplasm of kidney by 1.6% (OR = 1.02, 95% CI: 1.00–1.03, *P*-fdr = .02).

**Table 2:** The relationship between MSAB and kidney diseases in the offspring before removing offspring-related smoking SNPs.

Outcome	No. of SNP	Method	OR (95% CI)	*P*	*P*-fdr
Acute renal failure	15	Inverse variance weighted	13.18 (3.86–45.04)	<.001	<.001
		MR Egger	–	.66	.95
		Weighted median	6.93 (1.34–35.95)	.02	.05
		Weighted mode	4.04 (0.24–68.81)	.35	.44
Chronic kidney disease	16	Inverse variance weighted	2.82 (1.06–7.51)	.04	.05
		MR Egger	3.07 (0.01–847.61)	.70	.95
		Weighted median	3.38 (0.88–13.01)	.08	.09
		Weighted mode	4.78 (0.59–38.92)	.17	.30
Chronic renal failure	16	Inverse variance weighted	5.21 (1.76–15.44)	.003	.007
		MR Egger	163.81 (0.43–62103.49)	.12	.57
		Weighted median	6.32 (1.36–29.40)	.02	.05
		Weighted mode	12.32 (0.86–176.63)	.08	.30
Glomerulonephritis	16	Inverse variance weighted	3.82 (0.68–21.55)	.13	.13
2		MR Egger	0.32 (0.00–6739.72)	.83	.95
		Weighted median	8.24 (0.76–89.08)	.08	.09
		Weighted mode	12.32 (0.28–540.27)	.21	.30
Malignant neoplasm of kidney	6	Inverse variance weighted	1.02 (1.00–1.03)	.01	.02
		MR Egger	1.00 (0.90–1.10)	.95	.95
		Weighted median	1.01 (1.00–1.03)	.13	.15
		Weighted mode	1.01 (0.99–1.03)	.49	.52

SNP = single nucleotide polymorphism.

### 3.2. Association between MSAB and offspring kidney diseases (excluding smoking-related SNPs)

The MR analysis results (Table 3) indicated that, after excluding offspring-related smoking SNPs, MSAB led to a 10.47-fold increase in the incidence of acute renal failure in offspring (OR = 11.47, 95% CI: 2.58–51.02, *P*-fdr = .008). It also raised the risk of malignant kidney neoplasm by 2.3% (OR = 1.02, 95% CI: 1.01–1.04, *P*-fdr = .01). Nevertheless, after excluding offspring-related smoking SNPs, no statistically significant causal relationship was found between maternal smoking and CKD or chronic renal failure. The causal association between maternal smoking and glomerulonephritis (OR = 8.63, 95% CI: 1.13–65.88, *P*-fdr = .08) was no longer significant after adjusting the *P*-values using the FDR method.

**Table 3:** The relationship between MSAB and kidney diseases in the offspring after removing offspring-related smoking SNPs.

Outcome	No. of SNP	Method	OR (95% CI)	*P*	*P*-fdr
Acute renal failure	11	Inverse variance weighted	11.47 (2.58–51.02)	.001	.007
		MR Egger	–	.91	.91
		Weighted median	6.89 (1.04–45.44)	.05	.14
		Weighted mode	5.74 (0.38–87.43)	.24	.35
Chronic kidney disease	12	Inverse variance weighted	2.62 (0.83–8.29)	.10	.10
		MR Egger	9.87 (0.01–16075.54)	.56	.91
		Weighted median	2.22 (0.46–10.71)	.32	.34
		Weighted mode	1.49 (0.07–32.40)	.80	.80
Chronic renal failure	12	Inverse variance weighted	3.28 (0.90–12.01)	.07	.09
		MR Egger	10.35 (0.00–41113.64)	.60	.90
		Weighted median	3.11 (0.54–17.76)	.20	.25
		Weighted mode	4.77 (0.41–56.01)	.24	.35
Glomerulonephritis	12	Inverse variance weighted	8.63 (1.13–65.88)	.04	.06
		MR Egger	6.23 (0.00–2887296.95)	.79	.91
		Weighted median	8.50 (0.77–94.46)	.08	.16
		Weighted mode	10.34 (0.20–542.13)	.27	.35
Malignant neoplasm of kidney	4	Inverse variance weighted	1.02 (1.01–1.04)	.004	.01
		MR Egger	1.03 (0.92–1.15)	.63	.91
		Weighted median	1.02 (1.00–1.04)	.02	.08
		Weighted mode	1.02 (1.00–1.05)	.16	.35

CI = confidence interval, FDR = false discovery rate, MR = Mendelian randomization, MSAB = maternal smoking around birth, OR = odds ratio, SNP = single nucleotide polymorphism.

### 3.3. Sensitivity analysis results

Our sensitivity analyses included assessments of heterogeneity and tests for horizontal pleiotropy (Table 4). After after excluding offspring-related smoking SNP, we found no evidence of horizontal pleiotropy in any MR results, as all *P*-values from the MR Egger intercept analysis exceeded .05. Additionally, the heterogeneity analysis revealed no statistically significant heterogeneity (*P* > .05) in the MR results.

**Table 4:** Heterogeneity and horizontal pleiotropy in the present Mendelian randomization study.

Outcomes	Heterogeneity				Horizontal pleiotropy	
	*Q*	*Q*-df	*I* ^2^	*P*	Intercept in MR Egger regression	*P* (MR Egger intercept analysis)
Acute renal failure	3.371	10	0	.971	0.013	.71
Chronic renal failure	9.090	11	0	.614	-0.008	.79
Chronic kidney disease	7.292	11	0	.775	-0.010	.73
Glomerulonephritis	1.860	11	0	.999	0.002	.96
Malignant neoplasm of kidney	1.361	3	0	.715	-0.00005	.90

### 3.4. Multivariate analysis results

The TSMR analysis provided causal effect estimates derived from various MR methods. We observed that genetically predicted MSAB was significantly associated with the risk of acute renal failure (OR: 13.43, 95% CI: 2.72–66.25, *P*_IVW_ = .001), and malignant neoplasm of kidney (OR: 1.02, 95% CI: 1.01–1.04, *P*_IVW_ = .005) in offspring, but no causal relationship with other kidney diseases we studied.

## 4. Discussion

### 4.1. Key findings

This study examines how maternal smoking causes kidney diseases in offspring and highlights the importance of our findings. Our results show a strong link between maternal smoking during and after pregnancy and a higher risk of acute kidney failure and kidney cancers in offspring. There may also be a causal connection to glomerulonephritis. Importantly, even after accounting for factors like breastfeeding using advanced statistical methods, the association remained statistically significant. While the relative effect size (OR = 11.47) is substantial. It is important to note that the MR estimate represents the lifetime effect of genetic predisposition to the exposure, and its scale may not be directly comparable to the effect of short-term, modifiable exposure measured in observational studies. Therefore, it should not be interpreted as the immediate risk increase from a transient change in exposure level. Conversely, the effect size for renal malignancy (OR = 1.02) is modest in relative terms, but applied over a lifetime and across a population, it represents a non-negligible absolute risk increase. This underscores the importance of considering in utero exposures as part of a child’s long-term cancer risk profile. These findings highlight the serious impact of maternal smoking on the kidney health of future generations and emphasize the urgent need for public health interventions to reduce smoking during pregnancy.

Our study’s findings align with previous research indicating a strong association between maternal smoking and various adverse health outcomes in offspring, particularly concerning kidney diseases. A systematic review by Hwang et al showed that maternal smoking negatively affects the development of fetal kidneys, resulting in smaller size, volume, and function, especially for those exposed to more than 5 to 10 cigarettes daily.^[9]^ Numerous large population studies indicate that being small for gestational age and having low birthweight raise the risk of developing proteinuria, CKD, and kidney failure later in life.^[32]^ One possible explanation is that maternal smoking and alcohol consumption lead to low birthweight. This, in turn, results in smaller kidneys and fewer nephrons in children, potentially causing hyperfiltration and subsequent glomerulosclerosis, which increases vulnerability to kidney damage.^[33,34]^ Additionally, studies indicate that maternal smoking during pregnancy negatively impacts fetal development by allowing harmful substances to pass through the placenta.^[35]^ The placenta serves as a critical interface between the mother and fetus, facilitating the exchange of nutrients and waste. However, it can also transfer toxic substances, such as nicotine and other harmful chemicals found in cigarette smoke.^[36]^ Research shows that these substances can cross the placental barrier and enter fetal circulation, causing harmful effects on fetal organs, including the kidneys.^[37]^ An animal studies show that when mothers are exposed to nicotine during pregnancy and breastfeeding, their offspring may develop renal fibrosis, which is an irreversible effect of kidney injuries.^[38,39]^

Maternal smoking affects not only the structure of fetal kidneys but also has significant impacts on the development of renal blood vessels. Research indicates that higher doses of nicotine in cigarette smoke cause vasoconstriction, which is essential for normal kidney function.^[40]^ This impairment can lead to changes in kidney blood flow, ultimately affecting the kidney’s ability to filter blood effectively.^[41]^ Additionally, maternal smoking alters fetal development by affecting metabolic pathways that may increase cancer susceptibility later in life. These associations may stem from tobacco smoke’s effects on fetal deoxyribonucleic acid (DNA) methylation.^[42]^ A large-scale meta-analysis found a significant link between maternal smoking and differential methylation at 8862 cytosine-phosphate-guanine sites.^[43]^ This highlights the profound impact of maternal smoking on fetal DNA methylation. Rashmi et al found that gestational tobacco smoke exposure was linked to increased DNA methylation at the Paired Box Gene 8 (PAX8) (+5.22% average methylation; 95% CI: 0.33–10.10%; *P* = .037).^[44]^ This suggests that smoking during pregnancy may influence fetal health by affecting the methylation status of PAX8. PAX8 is a core regulatory factor continuously expressed in the thyroid and kidneys into adulthood.^[45]^ It activates hepatocyte nuclear factor 1 homeobox b via the enhancer conserved sequence, affecting downstream pathways such as cyclic adenosine monophosphate/phosphodiesterase 4C to regulate cell proliferation and differentiation. Hepatocyte nuclear factor 1 homeobox B plays a critical role in kidney function, and its abnormal expression is linked to polycystic kidney disease and renal cell carcinoma.^[46,47]^ Moreover, PAX8’s interaction with the wingless-related integration site (signaling pathway) (Wnt)/β-catenin signaling pathway underscores its significance in renal tubule formation and differentiation. The Wnt signaling pathway plays a crucial role in kidney development, and PAX8 promotes the formation and differentiation of renal tubules by regulating the activity of the Wnt signaling pathway.^[48]^ Harmful components in tobacco smoke can penetrate the placental barrier and disrupt fetal genomic stability through various mechanisms. Among them, the inhibition of DNA methyltransferase (DNMT) activity is a key molecular mechanism.^[49]^ Studies show that the absence of DNMT3A and DNMT3B in mouse models leads to abnormal expression of key genes during kidney development, thereby affecting the formation and function of nephron units. Therefore, maternal smoking during pregnancy is associated with changes in neonatal DNA methylation, which may persist into childhood and affect health outcomes.^[50]^

Maternal smoking may also lead to a decrease in the antioxidant capacity within the fetus, which increases the risk of oxidative damage during development.^[51]^ Research shows that smoking during pregnancy elevates oxidative stress and inflammation in the mother, which increases the risk of problems in fetal development, especially affecting kidney growth and function. Changes in oxidative stress and inflammation markers during pregnancy are closely linked to adverse pregnancy outcomes. This suggests that maternal smoking may affect fetal health by altering these biomarkers.^[52]^ Oxidative stress activates the nuclear factor kappa B signaling pathway, which in turn increases the expression and activity of transforming growth factor β. Activation of nuclear factor kappa B triggers the production of inflammatory and pro-fibrotic factors. These factors interact to form a positive feedback loop that promotes renal interstitial fibrosis.^[53]^ Moreover, maternal inflammatory factors like interleukin-6 can cross the placenta and directly affect fetal kidney development.^[54]^

In addition to direct developmental impacts, maternal smoking may also contribute to adverse health behaviors in offspring, including an increased likelihood of smoking initiation during adolescence. This intergenerational cycle of smoking behavior poses additional risks for kidney health, as smoking is a known risk factor for renal impairment and disease progression.^[50,55]^ The influence of maternal smoking extends beyond the immediate effects on fetal development, as it can shape the health trajectories of children as they grow into adulthood.

These findings provide a powerful evidence-based message for prenatal counseling: quitting smoking during pregnancy can potentially prevent severe, acute renal complications in the newborn and may also reduce the future risk of childhood kidney cancer. For neonates with significant in utero tobacco exposure, particularly those with other risk factors (e.g., low birth weight, sepsis), heightened clinical vigilance in the first 72 hours of life is warranted. Point-of-care biomarkers like urinary neutrophil gelatinase-associated lipocalin could serve as a valuable tool for early identification of subclinical renal injury before overt failure develops.^[56]^

From a public health perspective, this study strengthens the already compelling case for smoking cessation programs targeted at pregnant women and adolescents of reproductive age. The economic argument is reinforced: investing in such programs could not only improve birth outcomes but also prevent costly neonatal intensive care admissions for ARF and future oncology care. Our data could inform the development of targeted screening guidelines for high-risk infants, ensuring efficient use of healthcare resources.

### 4.2. Methodological strengths

This study presents a compelling causal association between maternal smoking and the risk of acute kidney failure and renal malignancies in offspring, which aligns with existing literature but enhances the reliability of these findings through a comprehensive multivariable MR approach. Our analysis rigorously adjusted for confounding factors like breastfeeding, unlike previous studies, which strengthens the validity of the observed associations. The application of multiple MR methodologies, including IVW, MR Egger, and WM approaches, further substantiates the robustness of our results. Moreover, sensitivity analyses, including tests for pleiotropy and heterogeneity, indicated no significant biases, adding confidence to our causal inferences. But the European-centric nature of our study imposes critical limitations on generalizability. Genetic architecture differences may attenuate effect sizes in non-European populations. The pooled prevalence of ever cigarette smoking in the continents of Oceania, Asia, Europe, America, and Africa was 36%, 14%, 38%, 31%, and 32%, respectively.^[57]^ Our conclusions are strictly constrained to populations of European ancestry until replicated in diverse cohorts. This research not only corroborates the adverse impacts of maternal smoking on renal health outcomes in offspring but also highlights the crucial role of methodological rigor in establishing reliable causal relationships, thereby guiding future public health interventions aimed at reducing maternal smoking during and after pregnancy.

### 4.3. Limitations

Despite the compelling results, this research has notable limitations. First, the GWAS summary data we extracted consisted solely of individuals of European ancestry, potentially leading to sample overlap. Thus, we need to confirm the results in other groups. we cannot address cross-ancestry differences without multi-ethnic data. This limitation reflects broader challenges in genetic epidemiology where 95% of GWAS data derive from European ancestry individuals.^[58]^ Limiting participants to European ancestry would mitigate the bias from population stratification, but it also limited the generalizability of our findings. Second, though multivariable MR adjusted for breastfeeding, residual confounding by unaccounted reproductive factors (e.g., gestational hypertension, preeclampsia) or socioeconomic determinants may persist. Third, our MR analysis results may be biased due to some potential pleiotropy. Nevertheless, the sensitivity analysis showed no significant effects caused by confounding biases. In conclusion, these findings highlight the urgent need for more clinical research to confirm these associations and investigate the biological mechanisms behind them. It is crucial to develop comprehensive public health strategies to address maternal smoking and mitigate its adverse effects on offspring kidney health.

## 5. Conclusion

This study establishes a strong causal link between maternal smoking and the risks of acute kidney failure and kidney tumors in offspring. While this aligns with existing literature, it enhances reliability through a comprehensive multivariable MR approach.^[59]^ The findings underscore the critical impact of maternal smoking on renal health outcomes in offspring, emphasizing the need for public health interventions aimed at reducing smoking during and after pregnancy.

## Acknowledgments

The authors sincerely thank the researchers and participants of the original GWASs for the collection and management of the large-scale data resources.

## Author contributions

**Conceptualization:** Shumin Kang.

**Data curation:** Zhenglong Zhang.

**Funding acquisition:** Shumin Kang, Weidong Xu.

**Investigation:** Zhenglong Zhang.

**Methodology:** Fengming Zhu, Jian Zhang.

**Project administration:** Weidong Xu.

**Resources:** Weidong Xu, Zhenglong Zhang.

**Software:** Fengming Zhu.

**Supervision:** Jian Zhang.

**Visualization:** Fengming Zhu.

**Writing – original draft:** Shumin Kang, Zhenglong Zhang.

**Writing – review & editing:** Shumin Kang, Fengming Zhu, Jian Zhang.

## Supplementary Material


