# Supplementary material for: The association between maternal smoking and the risk of kidney diseases in offspring: A Mendelian randomization analysis based on large-scale GWAS
Source: Medicine (Baltimore). 2025 Nov 7;104(45):e45711. doi: 10.1097/MD.0000000000045711 (PMC12599797; doi:10.1097/MD.0000000000045711)
Supplement: Supplementary file 1 [file medi-104-e45711-s001.docx]

**Table S1. Characteristics of the genetic variants associated with** **maternal smoking around birth （MSAB） and breastfeeding**

| MSAB / breastfeeding | SNP | Chr | Position | Effect allele | Other allele | Beta | SE | *P*-value | *F* |
| --- | --- | --- | --- | --- | --- | --- | --- | --- | --- |
| MSAB | rs10226228 | 7 | 32315613 | G | A | 0.007375 | 0.001063 | 4.00E-12 | 48.13721 |
| MSAB | rs12405972 | 1 | 44097438 | T | G | -0.00806 | 0.001075 | 6.40E-14 | 56.24693 |
| MSAB | rs12923476 | 16 | 24798079 | A | G | -0.00686 | 0.001173 | 4.80E-09 | 34.26019 |
| MSAB | rs1323341 | 9 | 14453010 | G | A | -0.00683 | 0.001243 | 3.90E-08 | 30.20328 |
| MSAB | rs2183947 | 6 | 26159356 | A | G | -0.00784 | 0.001225 | 1.50E-10 | 40.97711 |
| MSAB | rs2428019 | 11 | 113678423 | A | C | 0.007023 | 0.001202 | 5.10E-09 | 34.15846 |
| MSAB | rs35566160 | 2 | 164928199 | G | A | 0.006373 | 0.001165 | 4.50E-08 | 29.92673 |
| MSAB | rs36072649 | 4 | 140939110 | A | T | -0.00717 | 0.001057 | 1.10E-11 | 46.09363 |
| MSAB | rs4865667 | 5 | 50748173 | T | C | -0.00581 | 0.001053 | 3.40E-08 | 30.45095 |
| MSAB | rs576982 | 15 | 78870803 | T | C | -0.00933 | 0.001222 | 2.30E-14 | 58.27482 |
| MSAB | rs6011779 | 20 | 61984317 | T | C | -0.00994 | 0.001304 | 2.50E-14 | 58.08922 |
| MSAB | rs62477310 | 7 | 114951541 | C | T | -0.00578 | 0.00103 | 2.00E-08 | 31.49556 |
| MSAB | rs7002049 | 8 | 93114414 | C | T | 0.007562 | 0.00125 | 1.40E-09 | 36.6199 |
| MSAB | rs75596189 | 9 | 136468701 | T | C | 0.012053 | 0.001642 | 2.10E-13 | 53.86385 |
| MSAB | rs7899608 | 10 | 104727304 | T | C | 0.008782 | 0.001471 | 2.30E-09 | 35.66718 |
| MSAB | rs794356 | 7 | 75196531 | A | G | -0.00608 | 0.001082 | 1.90E-08 | 31.54569 |
| breastfeeding | rs1567820 | 18 | 37308349 | T | C | 0.006287 | 0.001121 | 2.00E-08 | 31.47334 |
| breastfeeding | rs2535296 | 6 | 31060219 | G | A | -0.00793 | 0.00138 | 9.10E-09 | 33.01494 |
| breastfeeding | rs56182580 | 7 | 132691858 | C | T | -0.00679 | 0.001195 | 1.30E-08 | 32.3093 |
| breastfeeding | rs8010613 | 14 | 42499198 | T | C | -0.0065 | 0.001164 | 2.40E-08 | 31.1616 |
| breastfeeding | rs9362076 | 6 | 85370492 | G | A | -0.00684 | 0.001204 | 1.30E-08 | 32.31309 |
| breastfeeding | rs9925536 | 16 | 8277742 | C | G | 0.006752 | 0.001141 | 3.20E-09 | 35.0438 |
